# Beyond the Capsid: How Can Post-Translational Modifications Modulate the Multifunctionality of the Orthoflavivirus Capsid Protein?

**DOI:** 10.3390/molecules31162901

**Published:** 2026-08-20

**Authors:** Nathane C. Mebus-Antunes, Dayane Henriques, Andrea T. Da Poian

**Affiliations:** Instituto de Bioquímica Médica Leopoldo de Meis, Universidade Federal do Rio de Janeiro, Rio de Janeiro 21941-902, RJ, Brazil; dayane.oliveira@bioqmed.ufrj.br

**Keywords:** capsid protein, post-translational modifications, orthoflavivirus, RNA virus

## Abstract

The orthoflavivirus capsid (C) protein is a multifunctional protein that plays essential roles throughout the viral life cycle. Besides viral RNA encapsidation for nucleocapsid assembly, it associates with lipid droplets, interacts with host proteins, and translocates to the nucleus, although its nuclear functions are still poorly understood. How these diverse activities are coordinated remains an open question. Post-translational modifications (PTMs), which are key regulators of protein function, have emerged as critical modulators of the infection cycle in many RNA viruses. However, little is known about the occurrence and functional significance of PTMs in orthoflavivirus C proteins. Here, we review the current evidence on PTMs in orthoflavivirus C proteins and integrate insights from studies of other RNA viruses to propose mechanisms by which PTMs may regulate C protein function. To complement this review, we performed a comparative in silico analysis of predicted PTM sites in the C proteins of dengue, Zika, West Nile, and Japanese encephalitis viruses. By integrating PTM predictions with experimentally validated modification sites, residue conservation, and structural mapping, we identified conserved regulatory hotspots that represent promising targets for future experimental validation. Together, these findings highlight PTMs as an underexplored regulatory mechanism in orthoflavivirus capsid biology and provide a framework for future mechanistic investigations.

## 1. Introduction

RNA viruses have small genomes and often encode a limited number of proteins. To ensure successful viral replication within the host, these proteins must perform multiple functions, acting at different stages of viral genome replication, as well as modulating cellular metabolism and host immune responses. Therefore, viral proteins are multifunctional [1,2,3].

*Orthoflavivirus*, a genus of positive-sense single-stranded RNA viruses within the *Flaviviridae* family, includes important human pathogens such as dengue virus (DENV), Zika virus (ZIKV), West Nile virus (WNV), Japanese encephalitis virus (JEV), yellow fever virus (YFV), and tick-borne encephalitis virus (TBEV). These viruses consist of enveloped icosahedral particles containing a small RNA genome of approximately 11 kb, which is directly translated into a single polyprotein. Subsequent processing of this polyprotein by viral (NS2B–NS3) and host (signal peptidase and furin) proteases yields ten viral proteins: three structural proteins (capsid, C; membrane, M; and envelope, E) and seven non-structural proteins (NS1, NS2A, NS2B, NS3, NS4A, NS4B, and NS5) [4]. Both canonical and non-canonical functions have been reported for each protein [2,3,5]. Table 1 summarizes the main functions reported for these proteins.

These multiple functions of viral proteins must be precisely regulated to ensure successful viral replication, with post-translational modifications (PTMs) representing one potential mechanism for regulating their multifunctionality [52,53]. Here, we review the PTMs described for orthoflavivirus C proteins and the capsid proteins of other RNA viruses, with particular emphasis on how PTMs may regulate these diverse functions.

## 2. How Do PTMs Modulate Viral Protein Functions?

PTMs are covalent chemical changes that occur after protein synthesis, primarily on amino acid side chains and at the N- and C-termini of proteins [54,55]. Major classes of PTMs include the addition of functional groups, such as phosphate, acetyl, and methyl groups (phosphorylation, acetylation, and methylation, respectively), as well as glycans (glycosylation), lipids (lipidation), and small proteins, including ubiquitin, SUMO, and other ubiquitin-like proteins. PTMs are generally catalyzed by enzymes, although some may also occur through non-enzymatic chemical reactions [54,55]. Many PTMs are dynamic and reversible, and their target sites are frequently enriched within intrinsically disordered regions (IDRs) or solvent-accessible regions of proteins [56,57]. Among amino acid residues, lysine is one of the most frequently modified, with its ε-amino group serving as a target for numerous PTMs [58].

By modifying the physicochemical properties of target amino acid residues, PTMs can influence protein conformation, dynamics, stability, hydrophobicity, charge, activity, localization, and interactions with other biomolecules [54,59]. Consequently, PTMs play essential roles in regulating numerous physiological processes and cellular responses to disease and infection, particularly during virus–host interactions [52,59]. Table 2 summarizes general information on the main PTMs.

During viral infection, PTMs can influence several stages of the viral life cycle, including viral entry, genome release, transcription, nucleocapsid (NC) assembly, and the degradation of viral proteins [52]. For example, acetylation of the NS3 protein contributes to viral replication in several orthoflaviviruses, including ZIKV, WNV, DENV, and YFV [70]. Similarly, acetylation at lysine 229 of the influenza A virus nucleoprotein (NP) is required for the efficient production of infectious viral particles [71]. Ubiquitination of the Ebola virus VP35 protein, which acts as an essential cofactor for the viral polymerase and as an antagonist of the host type I interferon (IFN-I) response, also facilitates viral polymerase activity and enhances viral replication [72]. In contrast, PTMs may also restrict viral infection. For instance, ubiquitination at lysine 653 of the influenza A virus polymerase subunit polymerase basic protein 1 (PB1) triggers its proteasomal degradation, thereby reducing polymerase activity [73]. Furthermore, previous studies have demonstrated the role of PTMs in regulating key stages of the host immune response and controlling essential host pathways required for an effective cellular antiviral response [52,74,75]. For example, TRIM25-mediated ubiquitination of RIG-I is essential for RIG-I signaling and contributes to the induction of the IFN-I response during infection with viruses such as Sendai virus and vesicular stomatitis virus (VSV) [76]. Similarly, SUMOylation of RIG-I promotes its ubiquitination and interaction with the downstream adaptor Cardif, thereby enhancing IFN-I signaling [77].

For orthoflaviviruses, several PTMs, including phosphorylation, ubiquitination, SUMOylation, acetylation, and glycosylation, have already been reported for structural and non-structural proteins [53,78]. However, only a few PTMs have been identified for the orthoflavivirus C protein. The canonical function of orthoflavivirus C proteins is genome packaging and NC assembly for the subsequent release of viral progeny [79,80]. Besides their roles in NC assembly, these proteins have additional functions in viral replication and host cellular responses (see Table 1), which are thought to depend, at least in part, on interactions with organelle membranes and protein partners [79,81]. The precise coordination of these diverse functions throughout the viral life cycle is likely mediated by multiple regulatory mechanisms, including PTMs.

## 3. PTMs in Orthoflavivirus Capsid Proteins: Current Knowledge

Capsid proteins are highly versatile and perform multiple functions beyond viral genome encapsidation [79,81]. Orthoflavivirus C proteins are small (~12 kDa), highly basic proteins composed of four α-helices (α1–α4), connected by short loops, and an N-terminal IDR of variable length among orthoflaviviruses [82,83]. In solution, these proteins form homodimers stabilized primarily by intermolecular interactions between the antiparallel α2/α2′ and α4/α4′ helices [82,83]. They contain approximately 25–27 basic residues (arginine (R) and lysine (K)), concentrated mainly in the N-terminal IDR and α4 helix, which confer a highly positive electrostatic surface potential [83]. The N-terminal IDR of DENVC is important for its association with membranes and lipid droplets (LDs) [84]. Its high density of positively charged residues has also been proposed to facilitate electrostatic interactions with viral RNA during encapsidation, which is suggested to be largely driven by charge neutralization [83,85,86]. However, the molecular mechanisms underlying this process remain poorly understood across orthoflaviviruses.

**Figure 1:**
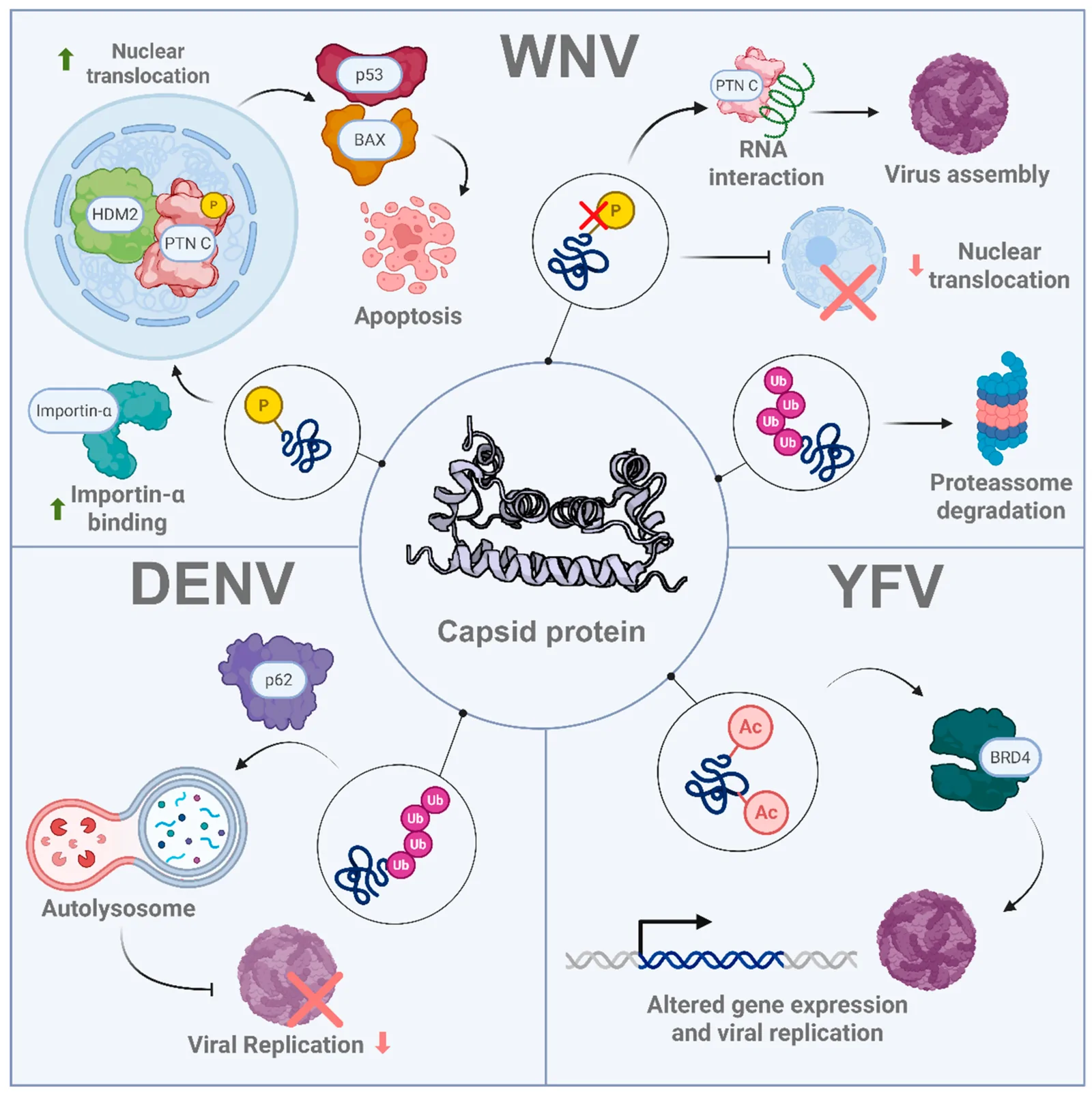
Overview of reported PTMs and potential PTM-associated regulatory mechanisms in orthoflavivirus C proteins. Schematic representation of experimentally identified PTMs in the C proteins of DENV, WNV, and YFV. The upper panel shows the WNVC representation. The lower section is divided into two side-by-side panels, showing the DENVC and YFVC representations, respectively. In WNVC, phosphorylation regulates nuclear localization, RNA binding, NC assembly, and apoptosis, whereas ubiquitination promotes proteasome-dependent degradation [8,87,88]. In DENVC, ubiquitination has been associated with interaction with the autophagy receptor p62 and autophagic degradation [89]. In YFVC, acetylation contributes to bromodomain protein 4 (BRD4) binding and modulation of host gene expression [90]. Arrows indicate enhancement or promotion of cellular events or interactions, whereas blunt-ended lines indicate inhibition or repression. Created in BioRender (https://BioRender.com/osmptu7 (accessed on 16 August 2026)).

The ability of orthoflavivirus C proteins to interact with multiple viral and host partners throughout the viral life cycle suggests that their diverse functions must be tightly coordinated in both space and time. Therefore, PTMs represent attractive candidates for regulating the multifunctionality of orthoflavivirus C proteins. To date, PTMs reported in orthoflavivirus C proteins are limited to phosphorylation in WNVC [8,87], ubiquitination in WNVC [88] and DENVC [89], and acetylation in the YFV C protein (YFVC) [90]. Figure 1 provides an overview of experimentally identified PTMs in orthoflavivirus C proteins, highlighting the limited number of modifications reported for these proteins to date.

### 3.1. Phosphorylation in Orthoflavivirus C Protein

Phosphorylation has been investigated primarily in WNVC. In this virus, phosphorylation has been shown to regulate functions such as NC assembly and apoptosis [8,87]. Phosphorylation has been identified at residues S83, S99, and T100 in studies performed in BHK cells. These residues are located within or adjacent to the nuclear localization signal (NLS) and are phosphorylated by protein kinase C (PKC) [8]. Mutation of these phosphorylation sites reduced WNVC binding to importin-α and increased its cytoplasmic localization [8]. Furthermore, the same study showed that WNVC phosphorylation enhances its binding to HDM2, a key negative regulator of the tumor suppressor p53, leading to increased levels of p53 and Bax, and activation of caspases-9 and -3, thereby triggering the p53-dependent apoptotic pathway [8]. In a subsequent study, the same group demonstrated that phosphorylation reduces WNVC binding to RNA and additionally investigated the roles of phosphorylation at S26 and S36 [87]. On the other hand, WNVC dephosphorylation increases RNA binding and reduces its nuclear translocation, allowing the protein to accumulate in the cytoplasm and interact with viral RNA, which may facilitate NC assembly [87].

### 3.2. Ubiquitination and Ubiquitin-like Modifications in Orthoflavivirus C Protein

Ubiquitination has been reported in both WNVC and DENVC and is primarily associated with protein degradation [88,89]. In WNVC, Makorin RING finger protein 1 (MKRN1) acts as an E3 ubiquitin ligase, targeting lysine residues K101, K103, and K104 [88]. Ubiquitination of WNVC leads to proteasome-dependent degradation of the protein and reduces WNV-induced cell death [88]. In DENVC, the ubiquitination at residue K76 has been identified as a possible recognition signal for binding to the host cargo receptor protein p62. This interaction directs DENVC to autophagic degradation and restricts viral replication [89].

A recent study showed that UFMylation, a ubiquitin-like modification, is important for orthoflavivirus infection. Disruption of the UFMylation machinery, particularly through depletion of the E3 ligase UFL1 in Huh7 cells during orthoflavivirus infection, reduces the production of infectious virus particles [91]. Furthermore, the same study identified that DENVC interacts with UFL1, raising the possibility that the UFMylation pathway contributes to the regulation of DENVC function [91].

### 3.3. Acetylation in Orthoflavivirus C Protein

Evidence for acetylation in orthoflavivirus C proteins is currently limited to YFVC [90]. The authors suggest that YFVC mimics histone H4 by harboring two acetylated lysine residues (K4 and K8) within a histone-like motif that interacts with bromodomain protein 4 (BRD4), thereby contributing to changes in host gene expression and viral replication. Whether acetylation plays additional roles in regulating orthoflavivirus C protein function remains to be determined.

Together, these findings suggest that distinct PTMs regulate different aspects of orthoflavivirus C protein functions, ranging from intracellular trafficking and RNA binding to protein turnover and host transcriptional regulation.

## 4. Potential Regulatory Roles of PTMs in Orthoflavivirus C Proteins

The study of PTMs in orthoflavivirus C proteins is particularly relevant because these proteins contain a high density of positively charged amino acid residues, with lysine and arginine residues concentrated in regions involved in protein–RNA and protein–membrane interactions [84,92,93]. This enrichment in target residues may make these regions more susceptible to PTMs. In particular, the structural flexibility of the N-terminal IDR is likely to increase its accessibility to modifying enzymes, making this region more accessible for modification [57]. Furthermore, C proteins’ IDRs are good candidates to interact with cellular components, thereby contributing to its diverse functions. Structural changes induced by PTMs, particularly within the IDR and the α4 helix, could regulate multiple events during viral replication, including dissociation from viral RNA following viral entry, intracellular localization, interactions with host factors, and recruitment of viral RNA during the assembly of new virions. Despite this potential regulatory role, PTMs that may alter the electrostatic interactions between the C protein and viral RNA, such as acetylation and methylation, remain largely unexplored.

### 4.1. In Silico Identification of Potential PTM Sites in Orthoflavivirus C Proteins

Understanding the role of PTMs in orthoflavivirus C proteins may help elucidate currently unknown aspects of viral replication and pathogenesis. To further explore this possibility, we analyzed the amino acid sequences of orthoflavivirus C proteins using prediction tools that employ different bioinformatics and computational approaches to identify putative PTM sites, including phosphorylation, methylation, acetylation, and SUMOylation [94,95,96,97,98,99,100,101,102,103,104,105]. Table S1 summarizes the prediction tools and their underlying computational strategies. The reference amino acid sequences of the C proteins from DENV (NP_739591.2), ZIKV (YP_009227196.1), WNV (NP_776010.1), and JEV (NP_775662.1) were submitted individually to each prediction tool.

The available prediction tools are primarily trained on eukaryotic protein datasets due to the limited availability of databases containing information on PTMs in viral proteins. Therefore, predictions obtained for viral proteins should be interpreted with caution and require experimental validation. To address this limitation, the viral PTM database (VPTMdb) web server was developed [101]. It combines the VPTMdb, which compiles experimentally validated PTMs from viral proteins and host proteins during viral infection, with VPTMpre, a prediction tool specifically designed to predict serine phosphorylation sites [101]. Analysis of the curated VPTM dataset indicates that most viral phosphorylation sites occur within consensus motifs shared with human proteins and that phosphorylation occurs preferentially within IDRs, consistent with observations in eukaryotic proteins [101]. VPTMpre provides three integrated machine learning-based prediction models (see Table S1). Among these, only the Naive Bayes model, a probabilistic machine learning classifier, predicted potential phosphorylation sites in the orthoflavivirus C proteins analyzed.

The integrated bioinformatics analyses identified several putative PTM sites in orthoflavivirus C proteins (Table 3). The predictions obtained for DENVC, ZIKVC, WNVC, and JEVC were evaluated according to the criteria established by each algorithm. For prediction tools that provide scores on a 0–1 scale, predictions with scores > 0.7 were considered high-confidence predictions. For phosphorylation predictions generated by GPS 6.0, residues were considered positive when the prediction score exceeded the corresponding kinase-specific cutoff. Kinase assignments predicted by GPS 6.0 and NetPhos 3.1 were not considered, and only the predicted phosphorylation sites were evaluated. To minimize false positives, candidate PTM sites were considered for inclusion if they met at least one of the following criteria: (i) were predicted by at least two independent prediction tools and occurred at homologous positions in at least two orthoflavivirus species; (ii) were predicted by at least one prediction tool and occurred at homologous positions in at least three orthoflavivirus species; or (iii) had been experimentally validated. Experimentally validated PTMs were included regardless of prediction status. Details of the predicted sites, prediction scores, and supporting tools are provided in Table S2.

**Figure 2:**
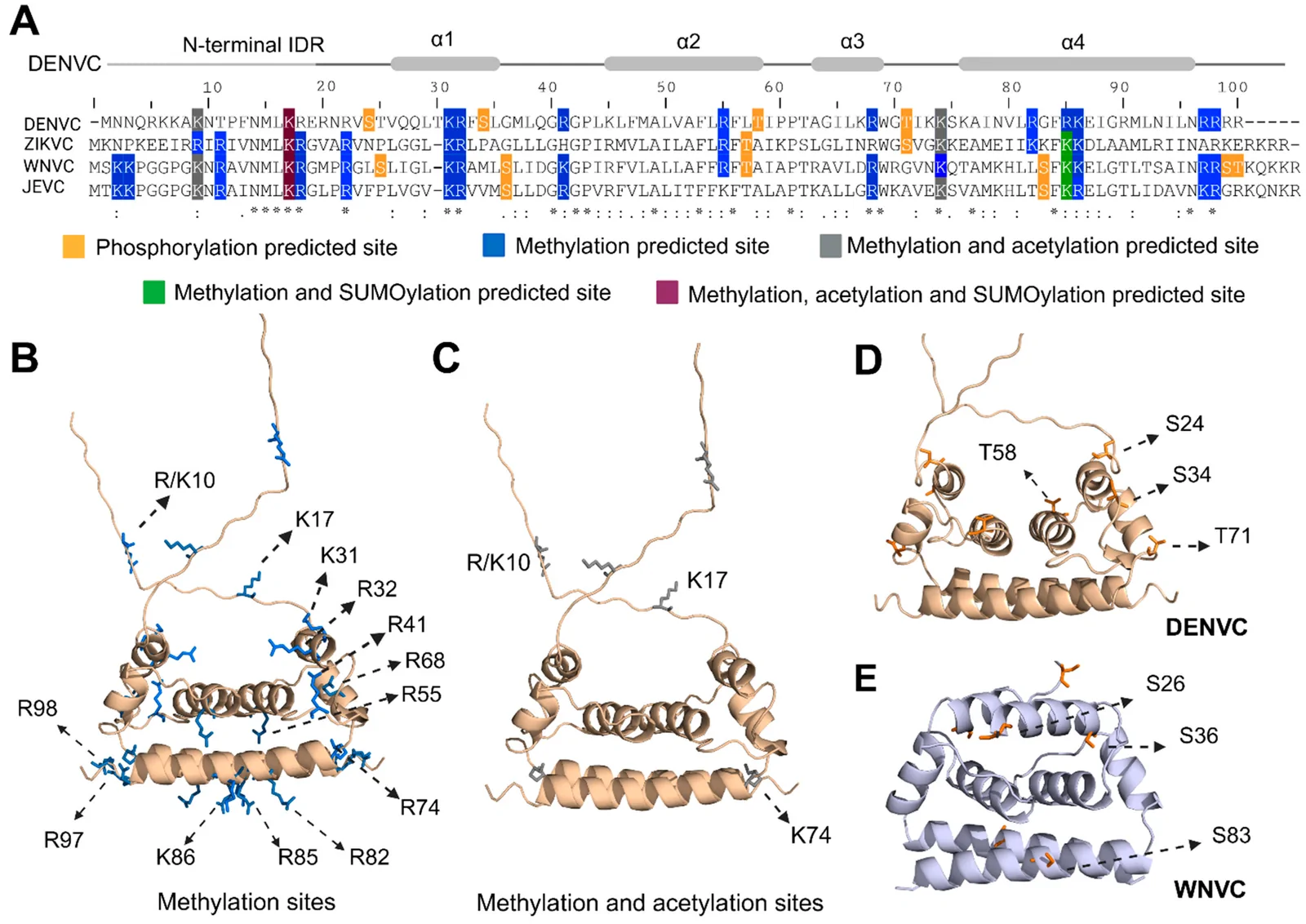
Predicted and experimentally validated PTM sites in orthoflavivirus C proteins. (**A**) Schematic representation of the secondary structure of the DENVC, showing the relative positions of the N-terminal IDR and the four α-helices (α1–α4). Multiple sequence alignment of orthoflavivirus C proteins generated using Clustal Omega v1.2.4 and visualized with Jalview v2.11.5.1. Predicted PTM sites are highlighted by color annotations corresponding to phosphorylation (orange), methylation (blue), methylation/SUMOylation (green), methylation/acetylation (gray), and methylation/acetylation/SUMOylation (purple). Clustal Omega conservation symbols (*, :, and .) are displayed below the alignment. (**B**–**D**) Structural representation of the DENVC (PDB: 1R6R, including a modeled N-terminal IDR) showing predicted PTM residues as sticks: methylation sites (blue) (**B**), methylation/acetylation sites (gray) (**C**), and phosphorylation sites (orange) (**D**). (**E**) Structural representation of the WNVC (PDB: 1SFK) showing experimentally validated phosphorylation sites S26, S36, and S83. Residues S99 and T100, which have also been experimentally identified as phosphorylation sites, are not shown because they are absent from the resolved structure. Protein structures were visualized using PyMOL v3.1.0. Created in BioRender (https://BioRender.com/ovzprvx (acessed on 17 August 2026)).

Conserved residues were identified based on a multiple sequence alignment of orthoflavivirus C protein sequences generated using Clustal Omega v1.2.4 [106] and visualized in Jalview v2.11.5.1 [107]. Homologous residue positions were defined according to the alignment (Figure 2A).

### 4.2. Predicted Phosphorylation Sites Across Orthoflavivirus C Proteins

Our analyses predicted all experimentally identified phosphorylation sites in WNVC (S26, S36, S83, S99, and T100) [8,87], demonstrating agreement between computational predictions and experimentally validated sites. Putative homologous phosphorylation sites were identified in JEVC (S36 and S83), whereas DENVC sites S24 and S34 were predicted near experimentally validated WNVC phosphorylation sites. Other predicted phosphorylation sites included T58 in DENVC, T57 in ZIKVC and WNVC, and T71 or S71 in DENVC and ZIKVC, respectively (Figure 2 and Table 3).

Notably, a recent study predicted phosphorylation sites and their corresponding kinases in DENV proteins using NetPhos 3.1, complemented by residue conservation analyses [108]. The phosphorylation sites predicted for DENVC were in good agreement with those identified in our analyses, further supporting the robustness of these predictions. Because several of these sites are conserved across orthoflaviviruses (Figure 2A), they represent promising candidates for future experimental investigation.

### 4.3. Potential Regulation of RNA Binding and NC Assembly by PTMs

Mutagenesis studies have demonstrated that mutations in conserved basic residues located within helices α1 (K31 and R32) and α4 (R85 and K86) attenuate infectious virus production in both DENV and JEV [93,109]. In our analyses, K31 and R32 were predicted to be methylated in all orthoflaviviruses analyzed, whereas K85 was predicted to undergo SUMOylation in ZIKVC, WNVC, and JEVC (see Table 3 and Figure 2). Thus, K31, R32, and K85 may represent key targets for PTMs that regulate C protein interactions with viral RNA and consequently influence NC assembly.

Besides these conserved basic residues involved in RNA interaction, additional putative PTM sites were identified, which may regulate C protein functions. SUMOylation typically occurs within the ψ-K-X-D/E consensus motif, where ψ represents a hydrophobic residue, K is the modified lysine, X is any amino acid, and D/E corresponds to aspartic or glutamic acid [110]. Our analysis predicted SUMOylation at K17 in DENVC, where this residue is located within the canonical SUMOylation consensus motif, and at the homologous K18 residue in the other analyzed orthoflaviviruses (Figure 2A). SUMOylation was also predicted at the homologous K85 position in ZIKVC, WNVC, and JEVC, where this lysine residue is located within a conserved SUMOylation consensus motif. In DENVC, the homologous position is occupied by R85 (Figure 2A).

Remarkably, the homologous K17/K18 site was predicted to undergo three different PTMs in all four orthoflaviviruses (see Table 3 and Figure 2). Located within the N-terminal IDR, K17/K18 lies in a region known to be particularly susceptible to PTMs [57]. The structural flexibility of the IDR likely promotes interactions with membrane structures, other proteins, and possibly viral RNA, making this region an attractive target for experimental investigation. The conserved K17/K18 site may represent a key regulatory site for the dynamic modulation of C protein functions and could contribute to elucidating how these proteins perform their roles during viral replication and NC assembly.

In addition to the N-terminal IDR, the α4 helix also contains residues that may be regulated by PTMs. Our recent studies suggest that the positively charged residues at positions 85 and 86 constitute an important region involved in viral RNA interaction and NC assembly [86,92]. Accordingly, PTMs within this region could alter its electrostatic properties, thereby affecting NC assembly or other stages of viral replication.

### 4.4. Potential Modulation of Intracellular Localization by PTMs

Subcellular localization of proteins can also be modulated by PTMs. Indeed, WNVC phosphorylation regulates its nucleocytoplasmic transport, a process that may also be regulated by PTMs in other orthoflaviviruses. Nuclear transport of DENVC involves three NLSs. Two of them correspond to the sequences KKAR (positions 6–9) and KKSK (positions 73–76), whereas the third is a bipartite NLS (RKEIGRMLNILNRRRR) located at positions 85–100, which is recognized by importin-α [111,112]. Mutagenesis studies in DENV-infected cells identified residues K73, K74, R85, and K86 as critical for nuclear localization in some cellular models [113]. Here, we identified K74, R/K85 and K86 as putative methylation sites, suggesting that these PTMs could be involved in DENVC nucleocytoplasmic trafficking. K74 is conserved among the four orthoflaviviruses analyzed and was predicted as a methylation site in all four viruses and as an acetylation site in three of them.

### 4.5. Potential Roles of PTMs in Membrane Association and Phase Separation

Interactions between C proteins and host lipid interfaces, particularly their association with ER-derived organelles such as lipid droplets (LDs), have been well described for DENVC, ZIKVC, WNVC, and JEVC [7,84,109,114,115]. For DENVC, this interaction, which is a crucial event for the formation of infectious DENV particles, is mediated by residues L50 and L54 located within the hydrophobic cleft of the α2 helix [7]. In addition to these residues, the DENVC N-terminal IDR is also involved in this association with LDs [84]. For ZIKVC, residues K31 and R32, located in the pre-α1 loop, appear to contribute to membrane association [115]. Interestingly, the PTM sites predicted in our analysis are concentrated within the N-terminal IDR and α1 region, which show variability among orthoflaviviruses (see Figure 2). Putative PTM sites were also identified within the α4 helix, a region known to be involved in key C protein functions [93].

PTMs can also have a significant impact on liquid–liquid phase separation (LLPS), a process involving the formation of biomolecular condensates, allowing cells to spatially and temporally coordinate biological processes [116]. For example, phosphorylation of the SARS-CoV-2 nucleocapsid (N) protein has been shown to modulate LLPS and the properties of the resulting condensates [117]. Interestingly, RNA binding has been shown to trigger LLPS during NC assembly in orthoflavivirus C proteins [118]. Given the growing evidence linking PTMs and LLPS during viral infection, investigating whether PTMs regulate LLPS in orthoflavivirus C proteins represents an important direction for future research.

### 4.6. Evolutionary Conservation and Structural Context of Predicted PTM Sites

Several of the residues identified here as putative PTM sites have previously been associated with important functions of the orthoflavivirus C protein. Features such as evolutionary conservation, surface exposure, and structural disorder provide additional evidence supporting the hypothesis that these residues represent potential PTM targets. To assess whether the predicted PTM sites displayed these characteristics, the sequences of the C proteins from the orthoflaviviruses analyzed were submitted to the ConSurf web server (https://consurf.tau.ac.il/, accessed on 5 August 2026) for residue conservation analysis.

**Figure 3:**
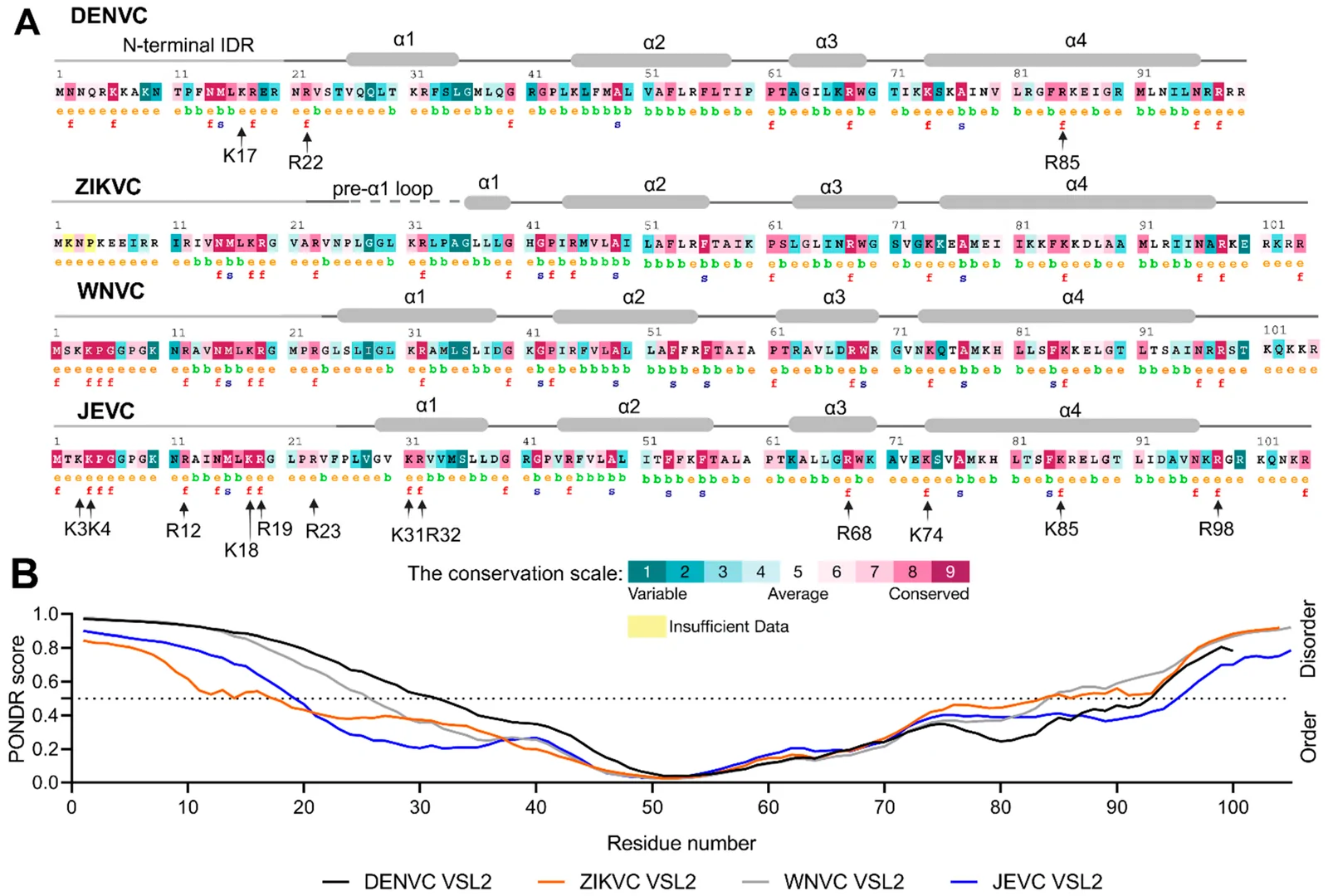
ConSurf and PONDR-VSL2 analyses of orthoflavivirus C proteins. (**A**) ConSurf conservation analysis combined with a schematic representation of the secondary structure organization of the orthoflavivirus C protein, showing the relative positions of the N-terminal IDR and the four α-helices (α1–α4) to facilitate the localization of the analyzed residues. Amino acid residues are colored according to their ConSurf conservation grades, ranging from turquoise (variable) to maroon (highly conserved). Residues with low-confidence conservation estimates are highlighted in light yellow. The annotations below each residue indicate ConSurf predictions: e, exposed residue (orange); b, buried residue (green); f, predicted functional residue (highly conserved and exposed) (red); and s, predicted structural residue (highly conserved and buried) (blue). ConSurf analysis was performed using FASTA sequences of DENVC, ZIKVC, WNVC, and JEVC. (**B**) Intrinsic disorder prediction was performed using PONDR VSL2 with the same protein sequences. The graph shows disorder propensity scores ranging from 0 to 1 for each residue, with values above 0.5 indicating intrinsically disordered regions. Black, DENVC; orange, ZIKVC; gray, WNVC; blue, JEVC. Created in BioRender (https://BioRender.com/svdyang (acessed on 17 August 2026)).

ConSurf identifies homologous sequences, constructs a multiple sequence alignment, and applies a Bayesian approach to estimate evolutionary conservation and identify residues with potential structural or functional importance [119]. Based on predicted solvent accessibility, residues are further classified as exposed or buried [119]. In addition, the sequences of the orthoflavivirus C proteins were analyzed using the PONDR-VSL2 server (http://www.pondr.com/, accessed on 4 August 2026) to predict intrinsic disorder propensity along the protein sequences [120]. Remarkably, among the predicted PTM sites, several residues were highly conserved, predicted to be surface-exposed and functionally important, and located within IDRs (Figure 3).

Within the N-terminal IDR, K3/K4 (WNVC/JEVC), R12 (ZIKVC/WNVC/JEVC), and the homologous R18/R19 positions were highly conserved and predicted to be functionally important (Figure 3A). The homologous R22/R23 and K31/R32 positions were also highly conserved, consistent with previous studies highlighting the functional importance of K31 and R32 (Figure 3A). Among the predicted PTM sites, the homologous K17/K18 positions emerged as one of the most notable candidates, as they were independently predicted as targets for methylation, acetylation, and SUMOylation across all analyzed orthoflaviviruses (Figure 3A). Within the α3 and α4 helices, R68, K74, and the homologous R/K85 positions were highly conserved and predicted to be functionally important (Figure 3A). Notably, K74 and R/K85 were also predicted as targets for multiple PTMs, highlighting the α4 helix as a potential regulatory hotspot.

PONDR-VSL2 predictions were consistent with the experimentally determined structural organization of orthoflavivirus C proteins [82,121,122], identifying the IDR and the structured α-helical domain (Figure 3B). Notably, VSL2 also predicted an increased tendency for disorder within the α4/α4′ region (Figure 3B). Most predicted PTM sites were concentrated within the N-terminal IDR and the α4/α4′, reinforcing that structurally flexible regions may represent preferential targets for regulatory PTMs (Figure 2 and Figure 3).

Collectively, the ConSurf and PONDR analyses indicate that several predicted PTM sites are evolutionarily conserved, surface exposed, and preferentially located within structurally flexible regions, providing additional support for prioritizing these residues for future experimental validation.

## 5. The Role of PTMs in Capsid Protein Functions of Other RNA Viruses

Studies of capsid proteins from other RNA viruses have revealed that PTMs represent important regulatory mechanisms that modulate diverse aspects of viral protein function. For nucleocapsid proteins of respiratory viruses, particularly SARS-CoV-2, PTMs are well documented and have been shown to modulate multiple functions, including protein oligomerization during NC assembly, viral RNA binding, and LLPS [123]. SUMOylation of the SARS-CoV-2 N protein has been associated with its homo-oligomerization and subcellular localization [110]. SUMOylation also contributes to the ability of the SARS-CoV-2 N protein to bind RNA, undergo LLPS, and suppress innate antiviral immune responses [124]. In addition to SUMOylation, other modifications, such as acetylation and methylation, also regulate the interaction of the SARS-CoV-2 N protein with viral RNA [125,126]. LLPS is also strongly influenced by PTMs; for example, phosphorylation of residues within the conserved serine–arginine (SR)-rich region is important for regulating this process [117]. Furthermore, phosphorylation at residues 188 and 206 within the SR-rich region promotes recruitment of the E3 ligase HERC5 and consequently induces N protein ISGylation, impairing N protein oligomerization and viral RNA synthesis [127].

PTMs of capsid proteins are particularly relevant for regulating viral replication, resulting in either pro- or antiviral effects. Methylation of RGG motifs in the SARS-CoV-2 N protein is important for viral particle production [126]. SUMOylation of the influenza A virus nucleoprotein (NP) alters its intracellular trafficking dynamics and contributes to efficient virus production [128]. Acetylation and ubiquitination of lysine residues in the influenza A virus NP have also been described as modifications that affect viral replication [71,129]. Conversely, the NP protein of influenza B virus undergoes ISGylation, which inhibits viral RNA synthesis by impairing NP oligomerization, resulting in an antiviral effect [130]. Ubiquitination of N proteins has been shown to mediate proteasomal degradation, as described for SARS-CoV-2, influenza A virus, and VSV [131,132,133]. Similarly, ubiquitination of the HCV core protein promotes its proteasomal degradation and has been associated with reduced protein availability for NC assembly [134].

Taken together, these studies demonstrate that PTMs in capsid proteins can regulate multiple steps of the viral life cycle, including RNA binding, NC assembly, intracellular trafficking, and antiviral responses. These findings further support the hypothesis that PTMs may play important regulatory roles in orthoflavivirus C protein functions. The major PTMs identified in nucleocapsid proteins of RNA viruses other than orthoflavivirus and their effects on the viral replication cycle are summarized in Table 4.

## 6. Conclusions and Future Perspectives

PTMs are major regulators of protein function in both physiological and pathological processes, including viral infections. Although their roles in orthoflavivirus structural and non-structural proteins have received increasing attention, little is known about their roles in capsid protein function. This review consolidates current knowledge and provides an integrated perspective on how PTMs may regulate key functions of the orthoflavivirus C protein. Despite the limited number of studies addressing PTMs in this protein, the available evidence highlights the need for further investigation through complementary in silico and experimental approaches, including the experimental validation of predicted PTM sites and the investigation of modifications that remain largely unexplored.

Future studies should pay particular attention to PTMs of positively charged residues, such as lysine and arginine, which are abundant in the capsid protein and are likely to play essential roles in viral RNA binding and NC assembly. We also identified several conserved candidate PTM sites, particularly within the N-terminal IDR, a structurally accessible region involved in membrane association and viral RNA interactions, making it an attractive target for regulatory modifications. As demonstrated for the capsid proteins of other RNA viruses, including SARS-CoV-2, the interplay among multiple PTMs may constitute a dynamic regulatory mechanism that fine-tunes protein function.

Recent advances in bioinformatics, artificial intelligence-based prediction tools, structural modeling, and molecular simulations have substantially improved the prediction and prioritization of candidate PTM sites for subsequent experimental validation [138,139]. However, experimental identification of PTMs remains challenging because these modifications are often of low abundance (frequently occurring at substoichiometric levels), transient, and chemically unstable. Moreover, the enzymes responsible for adding and removing PTMs act dynamically and are influenced by cell type and metabolic state, further complicating their experimental characterization. Despite these challenges, experimental validation remains essential to confirm predicted modification sites and establish their biological relevance.

Mass spectrometry-based proteomics remains the gold standard for PTM identification and site mapping, having contributed substantially to our understanding of the occurrence and dynamics of these modifications [140]. However, the high cost and the technical challenges associated with detecting low-abundance or transient modifications often limit its application. Complementary approaches, including antibody-based methods such as Western blotting and immunoprecipitation using modification-specific antibodies, provide valuable alternatives for validating individual PTMs [141]. Likewise, enrichment strategies, including immobilized metal affinity chromatography, ion-exchange chromatography, and immunoaffinity chromatography, facilitate the isolation of modified proteins or peptides, thereby increasing the sensitivity of downstream analyses [142]. Additional techniques, such as proximity ligation assays [143], protein microarrays [144], enzyme-linked immunosorbent assays [145], flow cytometry [146], and biorthogonal labeling approaches [147], further expand the experimental toolbox available for investigating PTMs in viral proteins.

The conserved candidate PTM sites identified in this review provide a valuable starting point for applying these complementary approaches. Combining computational predictions with targeted experimental validation will be essential not only to confirm individual PTM sites but also to determine how these modifications cooperate to regulate orthoflavivirus C protein functions throughout the viral replication cycle.

Together, the hypotheses we presented and conserved regulatory hotspots identified in this review provide a framework for future mechanistic studies aimed at elucidating how PTMs coordinate the diverse functions of orthoflavivirus C proteins. A better understanding of these regulatory mechanisms may not only advance our knowledge of orthoflavivirus biology but also reveal new opportunities for antiviral intervention.

## Figures and Tables

**Table 1:** Canonical and non-canonical functions of orthoflavivirus proteins.

Viral Protein	Canonical Functions	Non-Canonical Functions
C	Packages the viral genome and promotes nucleocapsid (NC) assembly [6].	Contributes to viral replication through nucleocytoplasmic trafficking and interactions with lipid droplets, nucleolar proteins, and other host factors, which can have either pro- or antiviral effects [7,8,9].
prM/M	prM: Acts as a chaperone for E protein folding and prevents premature fusion with the host membrane. M: Contributes to the structure and function of the mature virion [10,11,12].	Contributes to immune evasion by suppressing type I interferon (IFN-I) production [13]; contributes to ZIKV neurovirulence [14]; and enhances E protein immunogenicity in DENV [15].
E	Mediates receptor binding, viral entry through endocytosis, membrane fusion, and virion assembly [16,17].	Serves as the primary target of neutralizing antibodies, particularly through EDIII [18]. Also contributes to immune escape and antigenic variability [17,19]. Glycosylation of E protein influences viral production, pathogenicity, and neurovirulence [20,21].
NS1	Plays a critical role in viral RNA replication by interacting with other viral proteins and dsRNA to form the replication complex on ER-derived membranes together with NS4A and NS4B [22,23,24].	Contributes to immune evasion [25]; contributes to viral pathogenesis through interactions with host proteins, including complement components, the endothelial glycocalyx, and toll-like receptors [26,27]. NS1 has also been proposed as a diagnostic biomarker [28].
NS2A	Participates in viral RNA synthesis and is proposed to associate with viral RNA through interactions with the 3′UTR, as well as with C–prM–E complexes and NS2B–NS3 during virion assembly [29,30].	Suppresses IFN-β promoter-mediated transcription, thereby inhibiting the host innate immune response [31].
NS2B	Anchors NS3 to the ER membrane, forming the active NS2B–NS3 protease complex [32].	Antagonizes IFN signaling [33].
NS3	Acts as the viral protease, using NS2B as a cofactor, to cleave the viral polyprotein and some host proteins. Also exhibits helicase, nucleoside triphosphatase (NTPase), and RNA triphosphatase (RTPase) activities required for viral RNA replication and encapsidation [34].	Contributes to immune evasion [35,36]; interacts with and activates fatty acid synthase, influencing lipid metabolism [37]; impacts mitochondrial function [38]; and contributes to viral pathogenesis [39].
NS4A	Promotes ER membrane rearrangement required for formation of the viral replication complex [40].	Antagonizes IFN responses [41,42] and promotes autophagy [43] and mitophagy [44].
NS4B	Functions as a component of the replication complex through interactions with NS1, NS2B, NS3, and NS4A [45].	ZIKV NS4A and NS4B suppress host Akt-mTOR signaling, thereby impairing neurogenesis and inducing autophagy [46]. NS4B also antagonizes IFN signaling [47].
NS5	Functions as both the viral methyltransferase and RNA-dependent RNA polymerase and associates with NS2B and NS3 to form the replication complex on ER-derived membranes [48].	Antagonizes IFN signaling [49,50,51].

**Table 2:** Overview of the major PTMs and their biological functions.

Modification	Description	Target Residues	Enzymes	Functions
N-linked/O-linked glycosylation	Covalent addition of carbohydrate chains to protein molecules	Asparagine (N) (N-linked); serine (S) and threonine (T) (O-linked)	Glycosyltransferases (addition), glycosidases (removal)	Protein folding and stability, phase separation, cell adhesion, immune responses, cellular metabolism, and cell signaling [60].
ADP-ribosylation	Covalent addition of one or more ADP-ribose units to target proteins	Serine (S), threonine (T), tyrosine (Y), glutamate (E), aspartate (D), cysteine (C), arginine (R), lysine (K), and histidine (H)	ADP-ribosyltransferases	Regulation of cellular processes, stress responses, and metabolism [61].
Palmitoylation	Addition of a 16-carbon palmitoyl group to cysteine residues through thioester bonds	Cysteine (C)	Palmitoyltransferases (addition); depalmitoylases (removal)	Regulation of protein trafficking and stability; prevention of protein aggregation [62].
Phosphorylation	Addition of a phosphate group to amino acid side chains	Serine (S), threonine (T), or tyrosine (Y)	Kinases (addition), phosphatases (removal)	Signal transduction, regulating metabolism, cell growth, cell division, differentiation, motility, organelle trafficking, membrane transport, muscle contraction, and immunity [63].
Methylation	Addition of a methyl group to amino acid side chains	Lysine (K), arginine (R)	Lysine and arginine methyltransferases (addition); demethylases (removal)	Cellular signaling, DNA damage responses, protein stability, and protein–protein interactions [64,65].
Lysine acetylation	Covalent attachment of an acetyl group to lysine residues	Lysine (K)	Lysine acetyltransferases (addition); deacetylases (removal)	DNA binding and transcriptional regulation, protein–protein interactions, and protein stability [66].
Ubiquitination	Covalent attachment of ubiquitin, a 76-amino-acid protein, to target proteins	Lysine (K)	Ubiquitin-activating enzymes (E1), ubiquitin-conjugating enzymes (E2), ubiquitin ligases (E3); deubiquitinases (removal)	Proteasomal degradation, autophagy, cell signaling, endocytosis, receptor trafficking, DNA damage responses, cell cycle control, and programmed cell death [67].
SUMOylation	Covalent attachment of the small ubiquitin-like modifier (SUMO) to target proteins	Lysine (K)	SUMO-activating enzyme (SAE1/SAE2), SUMO-conjugating enzyme (Ubc9), SUMO ligases (E3); deSUMOylases (removal)	Cell cycle regulation, transcription, subcellular localization, protein stability, and chromatin organization [68].
ISGylation	Covalent attachment of the ubiquitin-like protein interferon-stimulated gene 15 (ISG15) to target proteins	Lysine (K)	ISG15-activating enzyme (UBA7), ISG15-conjugating enzyme (UBE2L6), ISG15 ligases (E3); deISGylases (removal)	Regulation of protein translation, autophagy, cytokine secretion, cytoskeletal dynamics, DNA damage responses, telomere shortening, innate immunity, including host defense against viral and bacterial infections [69].

**Table 3:** Comparative in silico prediction of PTM sites in orthoflavivirus C proteins, including consensus predictions across multiple tools and available experimental evidence.

Virus (C Protein)	Position	Residue(s)	PTM ^1^	Supporting Tools ^2,3^	Experimental Validation
WNV/JEV	3/3	K	Me	MeS; MS	-
WNV/JEV	4/4	K	Me	MeS; MS	-
DENV/ZIKV/WNV/JEV	9/10/10/10	K/R/K/K	Me	MeS; PTMG2	-
DENV/WNV/JEV	9/10/10	K	Ac	MD; PTMG2; DA	-
ZIKV/WNV/JEV	12	R	Me	PRme; CNN; PTMG2	-
DENV/ZIKV/WNV/JEV ^4^	17/18/18/18	K	Me	MeS; PTMG2	-
	17/18/18/18	K	Ac	DA; MD	-
	17/18/18/18	K	SUMO	SP; GS; MS	-
ZIKV/WNV/JEV	19	R	Me	PRme; CNN; PTMG2	-
ZIKV/WNV/JEV	23	R	Me	PRme; CNN; MS; PTMG2	-
DENV	24 ^5^	S	Phos	NP; GPS	-
WNV	26	S	Phos	NP; GPS; MS	[87]
DENV/ZIKV/WNV/JEV	31	K	Me	MeS	-
DENV/ZIKV/WNV/JEV	32	R	Me	PRme; CNN; PTMG2	-
DENV	34 ^5^	S	Phos	NP; MS; VPTM	-
WNV/JEV	36	S	Phos	NP	WNV [87]
DENV/WNV/JEV	41	R/K/R	Me	PRme; MeS; CNN; PTMG2	-
DENV/ZIKV/WNV	55	R	Me	CNN; PTMG2; DP	-
DENV/ZIKV/WNV	58/57/57	T	Phos	NP	-
DENV/WNV/JEV	68	R	Me	CNN; DP; PTMG2	-
DENV/ZIKV	71	T/S	Phos	NP; GPS; PTMG2	-
DENV/ZIKV/WNV/JEV	74	K	Me	MeS; PTMG2	-
DENV/ZIKV/JEV	74	K	Ac	PTMG2; DA	-
DENV/ZIKV	82	R/K	Me	PRme; CNN; MeS; PTMG2	-
WNV/JEV	83	S	Phos	NP; VPTM	WNV [8,87]
DENV/ZIKV/WNV/JEV	85	R/K/K/K	Me	CNN; MeS; PTMG2	-
ZIKV/WNV/JEV	85	K/K/K	SUMO	SP; GS; MS	-
DENV/ZIKV/WNV/JEV	86	K/K/K/R	Me	MeS; PTMG2; DP	-
DENV/WNV/JEV	97	R/R/K	Me	CNN; PTMG2; MeS	-
DENV/WNV/JEV	98	R	Me	PRme; CNN; PTMG2	-
WNV	99	S	Phos	NP; GPS	[8,87]
WNV	100	T	Phos	NP; GPS	[8,87]

^1^ PTM abbreviations: Ac, acetylation; Me, methylation; Phos, phosphorylation; SUMO, SUMOylation. ^2^ Supporting tools indicate the prediction programs that identified each candidate PTM site. Prediction scores and virus-specific predictions are provided in Supplementary Table S2. ^3^ Prediction tools abbreviations: MeS, MethylSight; PRme, PRmePRed; CNN, CNNArginineMe; MD, MDDeepAce; DA, DeepAcet; NP, NetPhos 3.1; GPS, GPS 6.0; VPTM, VPTMpre (Naïve Bayes model); SP, SUMOplot; GS, GPS-SUMO; DP, DeepPTMPred; MS, Musite; PTMG2, PTM-GPT2. ^4^ Sites associated with multiple PTM types were listed separately for each modification. ^5^ The S24 and S34 sites in DENVC were considered potential phosphorylation targets because they are located near experimentally validated phosphorylation sites in WNVC.

**Table 4:** Experimentally validated PTMs and their functional roles in RNA virus capsid proteins.

Viral Protein	PTM Site(s)	PTM	Biological Function ^1^
SARS-CoV-2 N protein	K61, K65 and K347	SUMOylation	K65 promotes NP oligomerization, RNA association, and LLPS; may contribute to innate immune suppression [124]. K65 is associated with nuclear translocation [110].
SARS-CoV-2 N protein	K375	Ubiquitination	Promotes N protein degradation via the proteasome [133].
SARS-CoV-2 N protein	K266, K355, K387, and K388	ISGylation	Regulates N protein oligomerization and viral RNA synthesis in a phosphorylation-dependent manner [127].
SARS-CoV-2 N protein	R95 and R177	Methylation	Promotes binding to the viral RNA 5′ UTR and viral particle production. R95 methylation also suppresses host stress granule formation [126].
SARS-CoV and SARS-CoV-2 N protein	K267 and K389 (SARS-CoV); K61, K100, K102, K237, K248, K249, K266, K355, K374, K375, K387, and K388 (SARS-CoV-2)	Acetylation	Proposed to affect RNA binding and interactions with other proteins [125].
SARS-CoV-2 N protein	SR-rich region (S188 and S206)	Phosphorylation	Modulates LLPS properties and is proposed to affect RNA binding and host protein localization to stress granules [117,135].
Influenza A virus NP	K4 and K7	SUMOylation	Regulates nucleocytoplasmic trafficking and contributes to efficient viral replication and virus production [128].
Influenza A virus NP	K77, K113, K184 and K229	Acetylation	K229 acetylation status influences the release of infectious virions [71].
Influenza A virus NP	K31, K77, K87, K91, K113, K184, K227, K229, K273 and K351 (CNOT4-mediated)	Ubiquitination	CNOT4-mediated ubiquitination promotes viral RNA replication [129]; TRIM41-mediated polyubiquitination promotes NP degradation [131].
Influenza B virus NP	K69, K77, K139, K156, K175, K245, K279, K294, K409 and K478	ISGylation	Inhibits NP oligomerization and impairs viral ribonucleoprotein formation and RNA synthesis [130].
Vesicular stomatitis virus NP	-	Ubiquitination	Promotes NP degradation via the proteasome, thereby limiting viral infection [132].
Hepatitis C virus core protein	-	Ubiquitination	Promotes proteasomal degradation of the core protein [134].
Hepatitis C virus core protein	S53 and S116	Phosphorylation	S116 phosphorylation may regulate nuclear localization of the core protein [136].
Hepatitis C virus core protein	C172	Palmitoylation	Associated with ER-associated LD targeting and efficient virion assembly and/or release [137].

^1^ Biological functions summarize experimentally demonstrated or proposed effects associated with the indicated PTM and are not necessarily attributable to all individual sites listed.

## Data Availability

The prediction data presented in the study are openly available in FigShare at 10.6084/m9.figshare.33283971.

## Supplementary Materials

The following supporting information can be downloaded at: https://www.mdpi.com/article/10.3390/molecules31162901/s1, Table S1: Overview of the PTM prediction tools used in this study. Table S2: Complete prediction results for PTM sites identified in orthoflavivirus C proteins by all prediction tools.
