# Supplementary material for: Beyond the Capsid: How Can Post-Translational Modifications Modulate the Multifunctionality of the Orthoflavivirus Capsid Protein?
Source: Molecules. 2026 Aug 20;31(16):2901. doi: 10.3390/molecules31162901 (PMC13515671; doi:10.3390/molecules31162901)
Supplement: Supplementary file 1 [file molecules-31-02901-s001.zip › molecules-4437720-supplementary.pdf]

Table S1. Overview of the PTM prediction tools used in this study.

Prediction tool	Website	PTM	Computational method	Training dataset	Ref.
PRmePRed	https://bioinfo.icgeb.res.in/PRmePRed/	Arg-Me	Physicochemical property encoding + Support Vector Machine (SVM)	UniProt/Swiss-Prot; PubMed literature	94
CNNArginineMe	https://github.com/guoyangzou/CNNArginineMe	Arg-Me	Convolutional Neural Network (CNN)	UniProt; PhosphoSite	95
MethylSight	https://methylsight.cu-bic.ca/	Lys-Me	ProtDCal feature extraction + Support Vector Machine (SVM)	PhosphoSite	96
MDDeepAce	https://github.com/Lxiaoyuleyuan/MDDeep-Ace	Lys-Ac	Multi-domain adaptation + CNN-LSTM	PLMD dataset (10 species)	97
DeepAcet	https://github.com/Lab-Xu/DeepAcet	Lys-Ac	Multilayer Perceptron (MLP) neural network	CPLM; UniProt	98
NetPhos 3.1	https://services.healthtech.dtu.dk/services/NetPhos-3.1/	Phos (S/T/Y)	Neural network	PhosphoBase, UniProt, PhosphoSite	99
GPS 6.0	https://gps.biocuckoo.cn/online.php	Phos (S/T/Y)	Ensemble of Deep Neural Networks (DNN) and Light Gradient Boosting Machine (LightGBM)	PubMed literature; EPSD; iEKPD; PDB	100
VPTMpre	http://vptmdb.com:8787/VPTMdb/	Phos (S/T/Y)	Support Vector Machine, Random Forest and Naïve Bayes	VPTMdb (PubMed literature, viral proteomics and UniProt/Swiss-Prot)	101
SUMOplot	https://www.abcepta.com/tools/SUMOplot	SUMO (Lys)	Consensus motif scoring based on motif matching and hydrophobic residue substitutions	—	—
GPS-SUMO	https://sumo.biocuckoo.cn/advanced.php	SUMO (Lys)	Transformer, Deep Neural Network and Penalized Logistic Regression	CPLM 4.0, dbPTM, qPTM, iPTMnet, PhosphoSitePlus, ProteomeScout,	102

				mUbiSiDa, HPRD, ActiveDriverDB, VPTMdb, PTMcode v2, UniProt, BioGRID and PubMed literature	
MusiteDeep	https://www.musite.net/	Multiple PTMs	Deep learning	UniProt/Swiss-Prot; RegPhos	103
DeepPTMPred	https://github.com/meilerlab/PTMPrediction/tree/main/data	Multiple PTMs	Hybrid CNN–Transformer deep learning framework	dbPTM	104
PTMGPT2	https://github.com/pallucs/PTMGPT2	Multiple PTMs	Prompt-based fine-tuned GPT-2 protein language model (decoder-only Transformer)	dbPTM; UniProt	105

Table S2. Complete prediction results for PTM sites identified in orthoflavivirus C proteins by all prediction tools.

Virus (C protein)	Position	Residue(s)	PTM[1]	Prediction tools (Score/Confidence) [2,3]
WNV/JEV	3/3	K/K	Me	MeS: 0.91 (WNV), 0.93 (JEV); MS: 0.83 (WNV), 0.83 (JEV)
WNV/JEV	4/4	K/K	Me	MeS: 0.92 (WNV), 0.94 (JEV); MS: 0.87 (WNV), 0.85 (JEV)
DENV/ZIKV/WNV/JEV	9/10/10/10	K/R/K/K	Me	MeS: 0.81 (DENV, 9K), 0.94 (WNV, 10K), 0.95 (JEV, 10K); PTMG2: 0.77 (DENV, 9K), 0.84 (ZIKV, 10R), 0.80 (JEV, 10K)
DENV/WNV/JEV	9/10/10	K/K/K	Ac	MD: 0.73 (DENV), 0.98 (WNV), 0.87 (JEV); PTMG2: 0.89 (DENV), 0.73 (WNV); DA: 0.85 (WNV), 0.84 (JEV)
ZIKV/WNV/JEV	12	R	Me	PRme: 0.97 (WNV), 0.96 (JEV); CNN: 0.76 (ZIKV), 0.85 (WNV), 0.81 (JEV); PTMG2: 0.78 (WNV)
DENV/ZIKV/WNV/JEV	17/18/18/18	K	Me	MeS: 0.84 (DENV), 0.74 (ZIKV), 0.94 (WNV), 0.93 (JEV); PTMG2: 0.82 (JEV)
DENV/ZIKV/WNV/JEV	17/18/18/18	K	Ac	DA: 0.78 (DENV), 0.69 (ZIKV)[4], 0.74 (WNV), 0.70 (JEV); MD: 0.75 (JEV)
DENV/ZIKV/WNV/JEV	17/18/18/18	K	SUMO	SP: 0.91 (DENV), 0.73 (ZIKV), 0.73 (WNV), 0.73 (JEV); GS: 0.85 (DENV), 0.77

				(ZIKV), 0.81 (WNV), 0.82 (JEV); MS: 0.83 (DENV)
ZIKV/WNV/JEV	19	R	Me	PRme: 0.94 (ZIKV), 0.95 (WNV), 0.92 (JEV); CNN: 0.95 (ZIKV), 0.93 (WNV), 0.89 (JEV); PTMG2: 0.87 (WNV), 0.89 (JEV)
ZIKV/WNV/JEV	23	R	Me	PRme: 0.83 (ZIKV), 0.97 (WNV); CNN: 0.82 (ZIKV), 0.94 (WNV), 0.88 (JEV); MS: 0.72 (WNV); PTMG2: 0.86 (JEV)
DENV	24	S	Phos	NP: 0.95; GPS: score 0.10 (cutoff 0.05)
WNV	26	S	Phos	NP: 0.95; GPS: score 0.98 (cutoff 0.02); MS: 0.81
DENV/ZIKV/WNV/JEV	31	K	Me	MeS: 0.80 (DENV), 0.84 (ZIKV), 0.92 (WNV), 0.92 (JEV)
DENV/ZIKV/WNV/JEV	32	R	Me	PRme: 0.70 (DENV), 0.95 (ZIKV), 0.88 (WNV), 0.72 (JEV); CNN: 0.82 (DENV), 0.77 (ZIKV), 0.77 (WNV), 0.70 (JEV); PTMG2: 0.76 (DENV), 0.87 (ZIKV), 0.92 (WNV)
DENV	34	S	Phos	NP: 0.90; MS: 0.85; VPTM: 0.94
WNV/JEV	36	S	Phos	NP: 0.99 (WNV), 0.99 (JEV)
DENV/WNV/JEV	41	R/K/R	Me	PRme: 0.98 (DENV, R), 0.97 (JEV, R); MeS: 0.76 (WNV, K); CNN: 0.90 (DENV), 0.95 (JEV, R); PTMG2: 0.87 (JEV, R)
DENV/ZIKV/WNV	55	R	Me	CNN: 0.76 (DENV); PTMG2: 0.87 (DENV), 0.79 (ZIKV), 0.72 (WNV); DP: 0.79 (ZIKV)
DENV/ZIKV/WNV	58/57/57	T	Phos	NP: 0.82 (DENV), 0.75 (ZIKV), 0.78 (WNV)
DENV/WNV/JEV	68	R	Me	CNN: 0.72 (WNV); DP: 0.94 (DENV); 0.80 (WNV); PTMG2: 0.85 (JEV)
DENV/ZIKV	71	T/S	Phos	NP: 0.98 (DENV, T), 0.96 (ZIKV, S); GPS: score 0.09 (cutoff 0.05, ZIKV, S); PTMG2: 0.77 (DENV), 0.77 (ZIKV)
DENV/ZIKV/WNV/JEV	74	K	Me	MeS: 0.88 (DENV), 0.88 (ZIKV), 0.77 (WNV), 0.80 (JEV); PTMG2: 0.85 (DENV), 0.80 (JEV)
DENV/ZIKV/JEV	74	K	Ac	PTMG2: 0.93 (DENV), 1.00 (ZIKV), 0.77 (JEV); DA: 0.73 (ZIKV)
DENV/ZIKV	82	R/K	Me	PRme: 0.95 (DENV, R); CNN: 0.97 (DENV, R); MeS: 0.92 (ZIKV, K); PTMG2: 0.71 (ZIKV, K)

WNV/JEV	83	S	Phos	NP: 0.95 (WNV), 0.99 (JEV); VPTM: 0.98 (WNV), 0.96 (JEV)
DENV/ZIKV/WNV/JEV	85	R/K/K/K	Me	CNN: 0.81 (DENV, R); MeS: 0.94 (ZIKV), 0.86 (WNV), 0.88 (JEV); PTMG2: 0.83 (DENV, R), 0.86 (WNV)
ZIKV/WNV/JEV	85	K/K/K	SUMO	SP: 0.85 (ZIKV);GS: 0.70 (ZIKV), 0.86 (WNV), 0.85 (JEV); MS: 0.75 (WNV)
DENV/ZIKV/WNV/JEV	86	K/K/K/R	Me	MeS: 0.93 (DENV, K), 0.93 (ZIKV, K), 0.84 (WNV, K); PTMG2: 0.79 (ZIKV, K), 0.77 (WNV, K); DP: 0.75 (WNV), 0.85 (JEV, R)
DENV/WNV/JEV	97	R/R/K	Me	CNN: 0.81 (DENV, R), 0.84 (WNV, R); PTMG2: 0.79 (WNV, R), 0.95 (JEV, K); MeS: 0.84 (JEV, K)
DENV/WNV/JEV	98	R	Me	PRme: 0.86 (JEV); CNN: 0.77 (DENV), 0.80 (JEV); PTMG2: 1.00 (ZIKV), 0.79 (WNV), 0.75 (JEV)
WNV	99	S	Phos	NP: 1.00; GPS: score 0.17 (cutoff 0.02)
WNV	100	T	Phos	NP: 0.96; GPS: score 0.06 (cutoff 0.02)

[1] PTM abbreviations: Ac, acetylation; Me, methylation; Phos, phosphorylation; SUMO, SUMOylation
[2] Prediction tools: MeS, MethylSight; PRme, PRmePRed; CNN, CNNArginineMe; MD, MDDeepAce; DA, DeepAcet; NP, NetPhos 3.1; GPS, GPS 6.0; VPTM, VPTMpre (Naïve Bayes model); SP, SUMOplot; GS, GPS-SUMO; DP, DeepPTMPred; MS, Musite; PTMG2, PTM-GPT2.
[3] Prediction scores >0.7 were considered indicative of high-confidence predictions. For GPS 6.0, residues were considered positive when the prediction score exceeded the corresponding kinase-specific cutoff (score ≥ cutoff). Kinase assignments predicted by GPS 6.0 and NetPhos 3.1 were not considered; only the predicted phosphorylation sites were evaluated.
[4] The acetylation site at ZIKVC K18 was retained despite a prediction score of 0.69 because the homologous position was predicted as an acetylation target in all four orthoflaviviruses and therefore fulfilled the conservation-based selection criterion.
